# Amino group in *Leptothrix* sheath skeleton is responsible for direct deposition of Fe(III) minerals onto the sheaths

**DOI:** 10.1038/s41598-017-06644-8

**Published:** 2017-07-26

**Authors:** Tatsuki Kunoh, Syuji Matsumoto, Noriyuki Nagaoka, Shoko Kanashima, Katsuhiko Hino, Tetsuya Uchida, Katsunori Tamura, Hitoshi Kunoh, Jun Takada

**Affiliations:** 1 Core Research for Evolutionary Science and Technology (CREST), Japan Science and Technology Agency (JST), Okayama University, Okayama, 700-8530 Japan; 2 Graduate School of Natural Science and Technology, Okayama University, Okayama, 700-8530 Japan; 3 Advanced Research Center for Oral and Craniofacial Sciences, Okayama University Dental School, Okayama, 700-8558 Japan; 4 Hyashibara Co., Ltd., Okayama, 702-8006 Japan

## Abstract

*Leptothrix* species produce microtubular organic–inorganic materials that encase the bacterial cells. The skeleton of an immature sheath, consisting of organic exopolymer fibrils of bacterial origin, is formed first, then the sheath becomes encrusted with inorganic material. Functional carboxyl groups of polysaccharides in these fibrils are considered to attract and bind metal cations, including Fe(III) and Fe(III)-mineral phases onto the fibrils, but the detailed mechanism remains elusive. Here we show that NH_2_ of the amino-sugar-enriched exopolymer fibrils is involved in interactions with abiotically generated Fe(III) minerals. NH_2_-specific staining of *L*. *cholodnii* OUMS1 detected a terminal NH_2_ on its sheath skeleton. Masking NH_2_ with specific reagents abrogated deposition of Fe(III) minerals onto fibrils. Fe(III) minerals were adsorbed on chitosan and NH_2_-coated polystyrene beads but not on cellulose and beads coated with an acetamide group. X-ray photoelectron spectroscopy at the N1s edge revealed that the terminal NH_2_ of OUMS1 sheaths, chitosan and NH_2_-coated beads binds to Fe(III)-mineral phases, indicating interaction between the Fe(III) minerals and terminal NH_2_. Thus, the terminal NH_2_ in the exopolymer fibrils seems critical for Fe encrustation of *Leptothrix* sheaths. These insights should inform artificial synthesis of highly reactive NH_2_-rich polymers for use as absorbents, catalysts and so on.

## Introduction

The biological strategies that living organisms use to produce a wide range of specially designed organic–inorganic materials such as bone, teeth, and shells^1^ have been exploited for artificially synthesizing materials for biomedical, industrial, and technological purposes and customizing their biomineral properties^1–5^. Of the many types of microbial sorbents (i.e., fungi, bacteria, and yeasts), some bacteria such as Fe/Mn-oxidizing bacteria frequently produce unique, ingenious structures with specific functions for their survival (e.g., to protect themselves, to stock and utilize nutrients, to move and function well) by biomineralization^6^. Not only the visual uniqueness of such structures but also their structural and physicochemical properties (e.g., exquisite organic–inorganic materials that are not readily synthesized artificially) have attracted researchers’ attention and provided many insights for developing and/or improving beneficial manufactured goods^7–10^.

*Leptothrix* species, common inhabitants of freshwater environments, oxidize Fe(II) in the presence of low oxygen concentrations^11–13^ and divide to form catenulate cells. They excrete exopolymer fibrils, which are entangled with interfibrillar cross-linkers such as proteins and disulfide bridges to eventually form a microtubular, immature, organic sheath skeleton^7, 14, 15^. Sugar analysis of sheath fibrils prepared from *L*. *cholodnii* SP-6 (hereafter referred to as SP-6) has elucidated that the basic structure of sheath fibrils comprise heteropolysaccharides containing galactosamine (GalN) and glucosamine (GlcN), both of which have a terminal NH_2_^16^. Another polysaccharide polymer with a terminal NH_2_, chitosan, is frequently used to adsorb transition metals from wastewater via the chelating ability of NH_2_ and an adjacent hydroxyl group^17–20^, suggesting that the terminal NH_2_ within the sheath fibrils might play a key role in metal encrustation.

The immature sheath skeleton of another *L*. *cholodnii* strain, OUMS1 (hereafter OUMS1-WT) becomes encrusted with metal cations and/or metal solid phases to eventually form uniquely structured microtubular sheaths comprising an organic–inorganic complex enriched with Fe, Si, P, and Ca^21–23^. Since an Fe(II)-oxidzing bacterium oxidizes Fe(II) and uses the generated electron as an energy source in the presence of low concentrations of oxygen^11–13^, the sheath probably possesses an ecological role in avoiding encrustation in Fe(III) oxyhydroxides. Fe(III) minerals (~50 nm diameter) that are generated abiotically in the culture medium (=complex of ferric oxyhydroxides as major components and inorganic components of the medium components as minors, Fig. S1) were reported to adhere directly to the sheath materials of SP-6^24^ and OUMS1-WT^25^. The Fe(III) minerals also directly adhere to cell- and protein-free sheath remnants, indicating that living cells and their proteins conjugated to sheath fibrils are not inevitably required for the Fe(III) mineral encrustation^24, 25^. The metal encrustation of the sheath skeleton is considered to result from the interactions between aquatic phase inorganic cations and the functional groups in the sheath skeleton^12^. Indeed, a strong correlation exists between the presence of acidic polysaccharides with carboxyl groups (COOH) and the distribution of iron oxyhydroxides in *Leptothrix* sheaths^6, 26^, but little is known about the roles of NH_2_ within the sheath fibrils on the metal encrustation.

**Figure 1:**
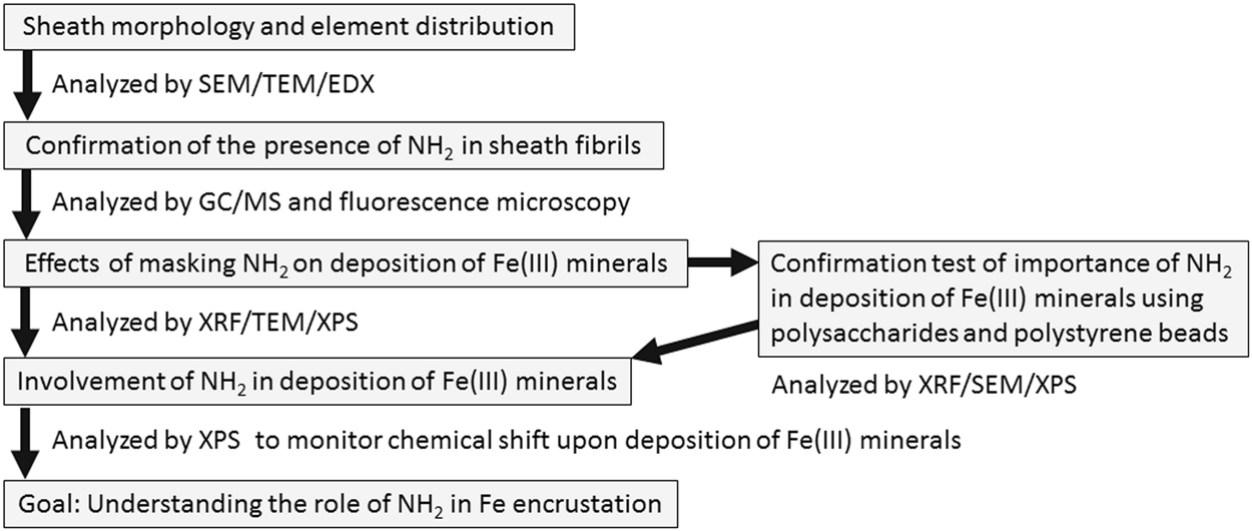
Flow chart of experiments toward the goal of this study.

In this study, we sought to ascertain the involvement of the terminal NH_2_ within the constitutive molecules of the immature sheath skeleton of OUMS1-WT in the adsorption of Fe(III) minerals to the skeleton by differential interference contrast (DIC) and fluorescent microscopy, scanning, transmission, and scanning-transmission electron microscopy (SEM, TEM, and STEM, respectively), energy-dispersive x-ray microanalysis (EDX), x-ray fluorescence and photoelectron spectroscopy (XRF and XPS, respectively) (Fig. 1).

## Results

### Importance of sheath fibrils for Fe encrustation

We examined the ultrastructure of OUMS1-WT grown in silicon-glucose-peptone medium (Table S1) (hereafter SGP)^27^ for 2 days and then in SGP + Fe-plate medium (hereafter SGP + Fe plate) for 2 more days for comparison with previous TEM images of the cells incubated in SGP + Fe plate for 3 days^28^. For comparison, a sheathless mutant of OUMS1 (named hereafter OUMS1-SL)^28^ was incubated and studied in similar conditions.

**Figure 2:**
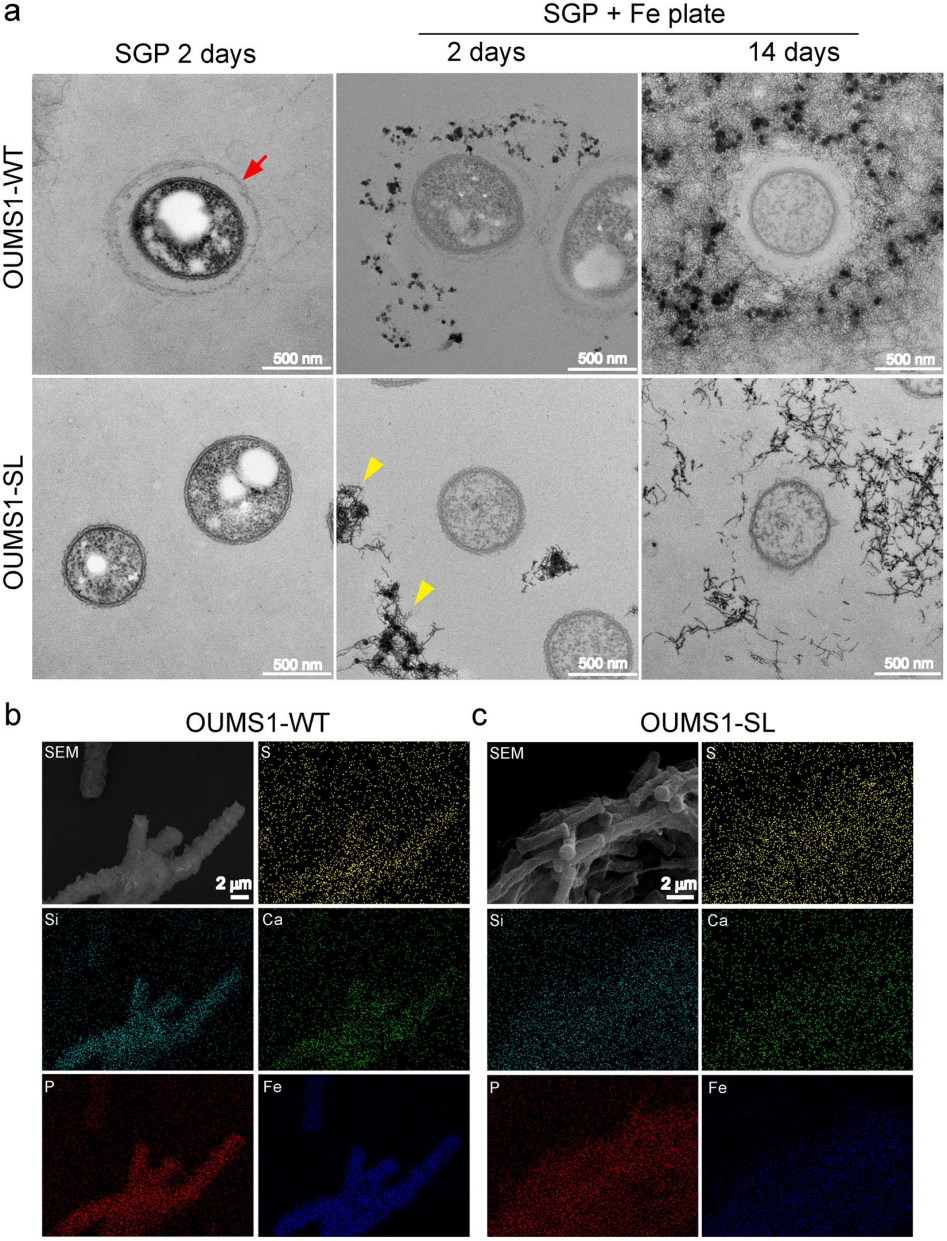
TEM images (**a**) of OUMS1-WT and OUMS1-SL after incubating in SGP and then with SGP + Fe plate and SEM images with EDX distribution patterns of elements (**b**) of both strains. (**a**) Left, SGP incubation for 2 days: cross section of OUMS1-WT cell encased with a thin, immature sheath (arrow) (upper left) and OUMS1-SL cell with no sheath (lower left). Center, additional 2-day incubation in SGP + Fe plate: electron-dense Fe particles on and near the immature sheath encasing OUMS1-WT cells (upper center) and on a few fibril aggregations far from OUMS1-SL cells (lower center, arrowhead). Right, additional 14-day incubation in SGP + Fe plate: deposition of electron-dense Fe particles in a thick sheath encasing OUMS1-WT cells and in abundant fibrils extending outward from the sheath surface (upper right). Electron-dense fibril aggregation seen apart from the OUMS1-SL cell (lower right), but no sheath has formed. (**b**) Aggregated immature sheaths of OUMS1-WT after incubating in SGP + Fe plate for 14 days with EDX distribution patterns of S, Si, Ca, P, and Fe showing apparent deposition of Si, P, and Fe on sheaths. (**c**) Aggregated chains of cells of OUMS1-SL after incubating in SGP + Fe plate for 14 days with EDX distribution patterns of S, Si, Ca, P, and Fe, lacking any distinguishable deposition, even of Fe.

After 2 days incubation in SGP, chains of OUMS1-WT cells (ca 3–4 μm long) were encased within a thin, immature sheath, while longitudinally extending chains of OUMS1-SL cells (ca 7–8 μm long) were not (Fig. 2a, left). After another 2 days incubation in SGP + Fe plate, a few aggregated fibrils were seen around OUMS1-SL cells, suggesting fibril excretion is enhanced in the presence of the Fe source, but they did not assemble into a sheath (Fig. 2, lower center) as reported previously^28^. Electon-dense particles were deposited on and near the immature sheath encasing OUMS1-WT cells (Fig. 2a, upper center) and on aggregated fibrils far from OUMS1-SL cells (Fig. 2a, lower center). To confirm the absence of sheaths in OUMS1-SL, the culturing period of both isolates in SGP + Fe plate was extended to 14 days. The OUMS1-WT cells were evidently encased within thick sheaths having lots of hairy fibrils extending outward onto which electron-dense particles deposited (Fig. 2a, upper right). Aggregated fibrils 10–150 μm from the OUMS1-SL cell became electron-dense but never assembled into a sheath (Fig. 2a, lower right).

SEM/EDX analyses of OUMS1-WT cells incubated in SGP + Fe plate as above detected a mass of aggregated sheaths and apparent deposition of Si, P, and Fe on sheaths (Fig. 2b), while chained OUMS1-SL cells aggregated without being encased in sheaths and lacked any distinguishable element depositon, even of Fe (Fig. 2c).

From these microscopic results, we confirmed that (i) OUMS1-WT formed immature thin sheaths within 2 days in SGP, while exopolymer fibrils were not excreted from OUMS1-SL cells, and no sheath formed; (ii) OUMS1-SL cells excreted fibrils but failed to form sheaths even after an additional 2 days incubation in SGP + Fe; and (iii) inorganics including Fe were deposited on OUMS1-WT sheaths within another 2 days in SGP + Fe plate, while Fe was not deposited on chained OUMS1-SL cells, which lacked a sheath.

### NH_2_ functional groups in the amino sugars of the OUMS1-WT sheaths play a role in Fe(III) mineral deposition

**Figure 3:**
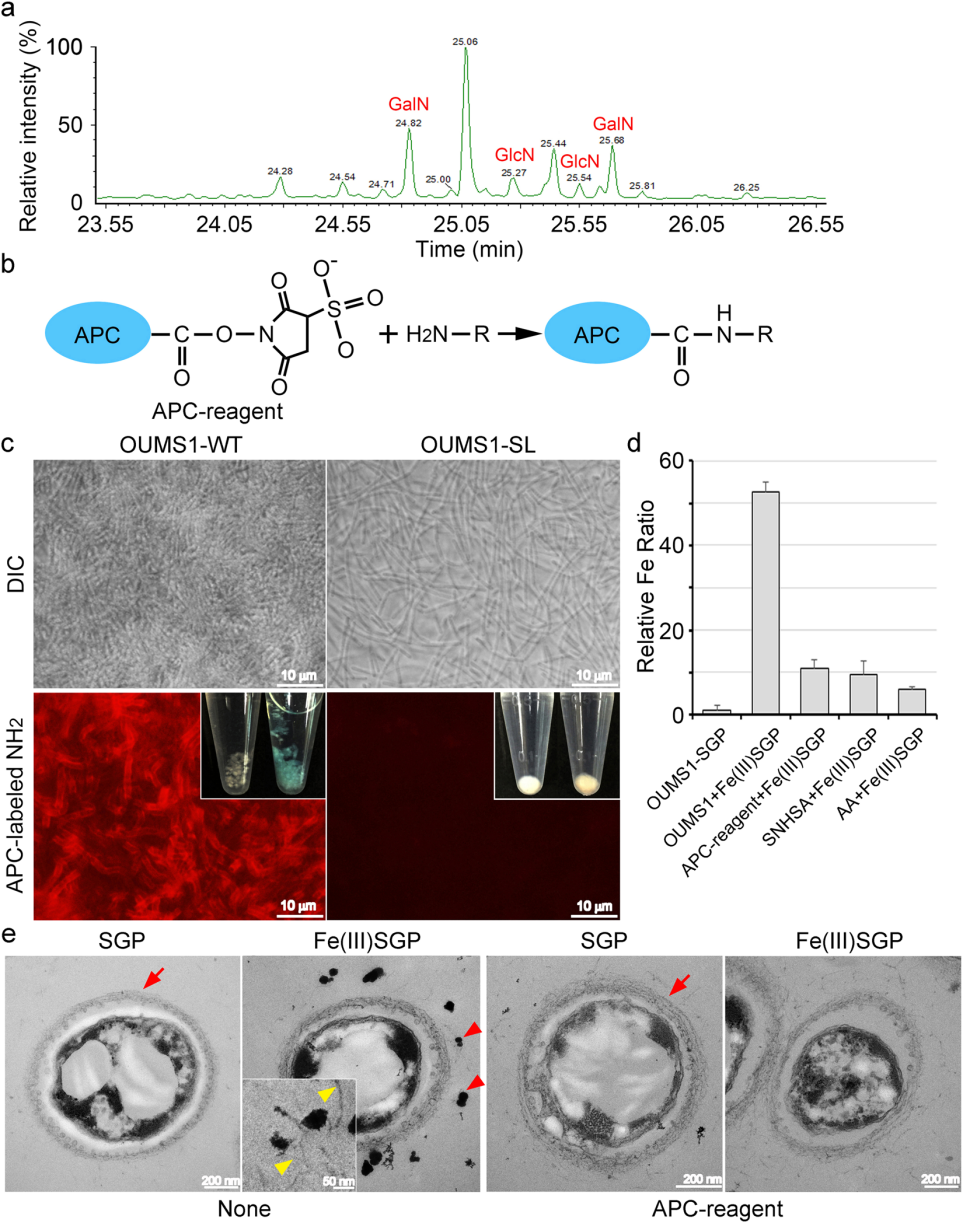
GC/MS spectrograph showing sugar composition in protein-free OUMS1-WT sheath remnants and attachment of Fe(III) minerals on OUMS1-WT sheath skeleton treated with NH_2_-binding fluorescent reagents. (**a**) GalN and GlcN were detected in the sheath remnants as major saccharic materials. (**b**) Schematic figure of allophycocyanin-conjugated NH_2_-reactive reagent (APC-reagent) masking NH_2_ group. (**c**) Top, DIC images of immature sheaths encasing OUMS1-WT cell chains and longitudinally extending, sheath-uncovered OUMS1-SL cell chains after treatment with APC-reagent. Bottom, APC-reagent-treated OUMS1-WT sheaths turned blue within 30 min (inset) and fluoresced red; similarly treated OUMS1-SL were visibly unchanged (inset) and did not fluoresce. (**d**) Fe ratios in OUMS1-WT sheaths and those treated with APC-reagent, sulfo-NHS-acetate, or acetic anhydride after incubation in Fe(III)SGP relative to those in Fe-free SGP (with At% of Fe = 1). Note the suppressed Fe levels in these NH_2_-masked sheaths. (**e**) TEM images of OUMS1-WT cells. None (APC reagent-untreated) 1^st^, a cell encased with thin, immature sheath (arrow) after incubation in SGP; 2^nd^, electron-dense Fe(III) minerals (arrowheads) attached to surface of immature sheath and fibrils extending from the surface after incubation in Fe(III)SGP (inset, enlarged image showing mineral attachment to the extending fibrils [arrowhead]). APC reagent 3^rd^, APC-reagent-treated cell encased with a thin, immature sheath (arrow) incubated in SGP; 4^th^, no Fe minerals on or near the sheath even after incubation in Fe(III)SGP.

We examined whether amino sugars were components of OUMS1-WT sheaths as found for SP-6^15, 16^ by using protein-free sheath remnants that had been chemically prepared from immature sheaths of OUMS1-WT cultured in SGP for 2 days (Fig. 3a). The GC/MS analysis revealed that GalN and GlcN were major saccharic components of the sheath fibrils of OUMS1-WT, as NH_2_-holding materials in addition to amino acids, similar to those in the *L*. *cholodnii* SP-6 sheath fibrils^15, 16^.

**Figure 4:**
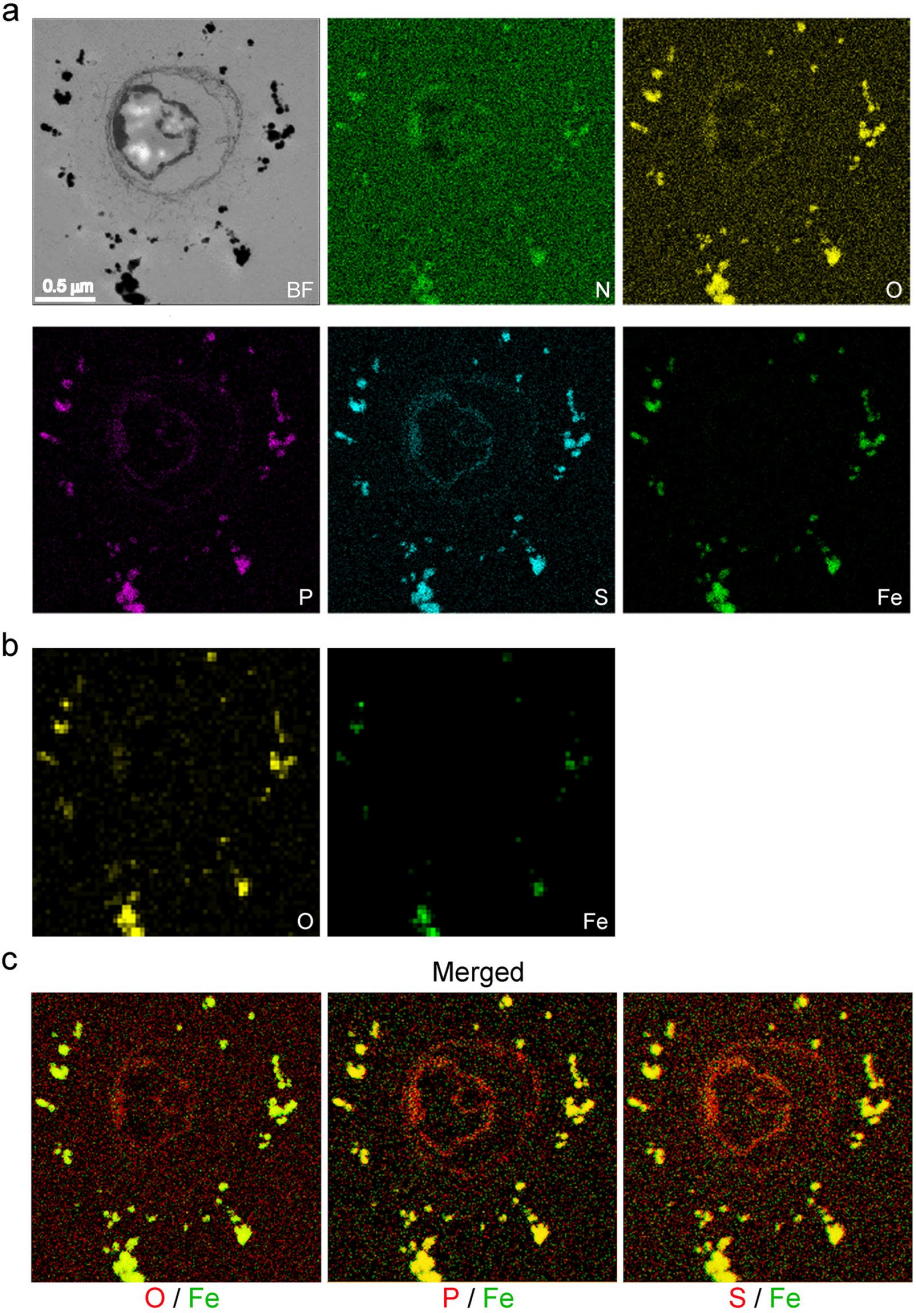
STEM image and EDX element distribution pattern on immature sheaths encasing OUMS1-WT cell incubated in Fe(III)SGP. (**a**) Top, STEM image of electron-dense particles attached to the sheath and distribution patterns of N and O. Bottom, distribution of P, S, and Fe. (**b**) Semiquantitative distribution maps of O and Fe determined by the Cliff-Lorimer method. The signals for both elements at the same location strongly suggest that Fe could exist as iron oxides. (**c**) Merged images for distribution of O/Fe, P/Fe, and S/Fe, suggesting that the electron-dense Fe particles plausibly bind the medium components.

Whitish, immature sheaths encasing OUMS1-WT cells that had been incubated in SGP for 2 days were treated with allophycocyanin (APC)-conjugated NH_2_-reactive reagent (hereafter APC-reagent) (Fig. 3b). Within 3 h, the OUMS1-WT sheaths turned visibly blue and, viewed with fluorescence microscopy, fluoresced an intense red (Fig. 3c, lower left), demonstrating the presence of terminal NH_2_ in the sheaths. In comparison, cells of OUM1-SL, incubated in SGP and similarly treated with the APC-reagent, remained white and did not fluoresce (Fig. 3c, lower right). The lack of excretion of exopolymer fibrils from OUMS1-SL cells within 2 days incubation in SGP (Fig. 2a) accounted for the lack of a positive response (change in color) or any notable deposition of elements such as Fe (Fig. 2c). Subsequently, the APC-reagent-treated OUMS1-WT sheaths were incubated with a suspension of abiotic Fe(III) minerals in SGP [hereafter Fe(III)SGP] to examine the influence of APC-masked NH_2_ in the sheath skeleton on Fe(III) mineral encrustation (Fig. 3b). XRF analysis of washed and freeze-dried untreated control sheaths revealed that the ratio of Fe was nearly 50 times higher in Fe(III)SGP relative to an atomic (At) % of Fe = 1 in Fe-free SGP (Fig. 3d). However, in the APC-reagent-treated sheaths, the relative ratio was enhanced approximately 10-fold more than after the SGP incubation (Fig. 3d). Similarly, the increase in Fe percentage after the Fe(III)SGP incubation was suppressed by two other NH_2_-blocking reagents, sulfo-NHS-acetate or acetic anhydride (Figs 3d and S2a,b). Electron-dense particles were apparently deposited around the immature control sheaths after incubation in Fe(III)SGP (Fig. 3e, left two), while such particles were not seen on or around APC-reagent-, sulfo-NHS-acetate-, or acetic anhydride-treated OUMS1-WT cells (Fig. 3e, right two, S2a,b). To confirm whether the deposited electron-dense particles corresponded to Fe minerals, we incubated the SGP-precultured OUMS1-WT cells with Fe(III)SGP for 2 days. EDX analyses of cross-sectioned cells (Fig. 4a,b) showed that the electron-dense particles on or around the exopolymer fibrils were composed of Fe, bound to the medium components such as P and S, as judged by the similar distribution of Fe/O and Fe/P/S (Fig. 4c). The XRD analysis provided evidence that the Fe detected by EDX was actually Fe(III) oxyhydroxide (Fig. S1a–c).

### Binding energy shift on the sheath surface caused by incubation with Fe(III)SGP

**Figure 5:**
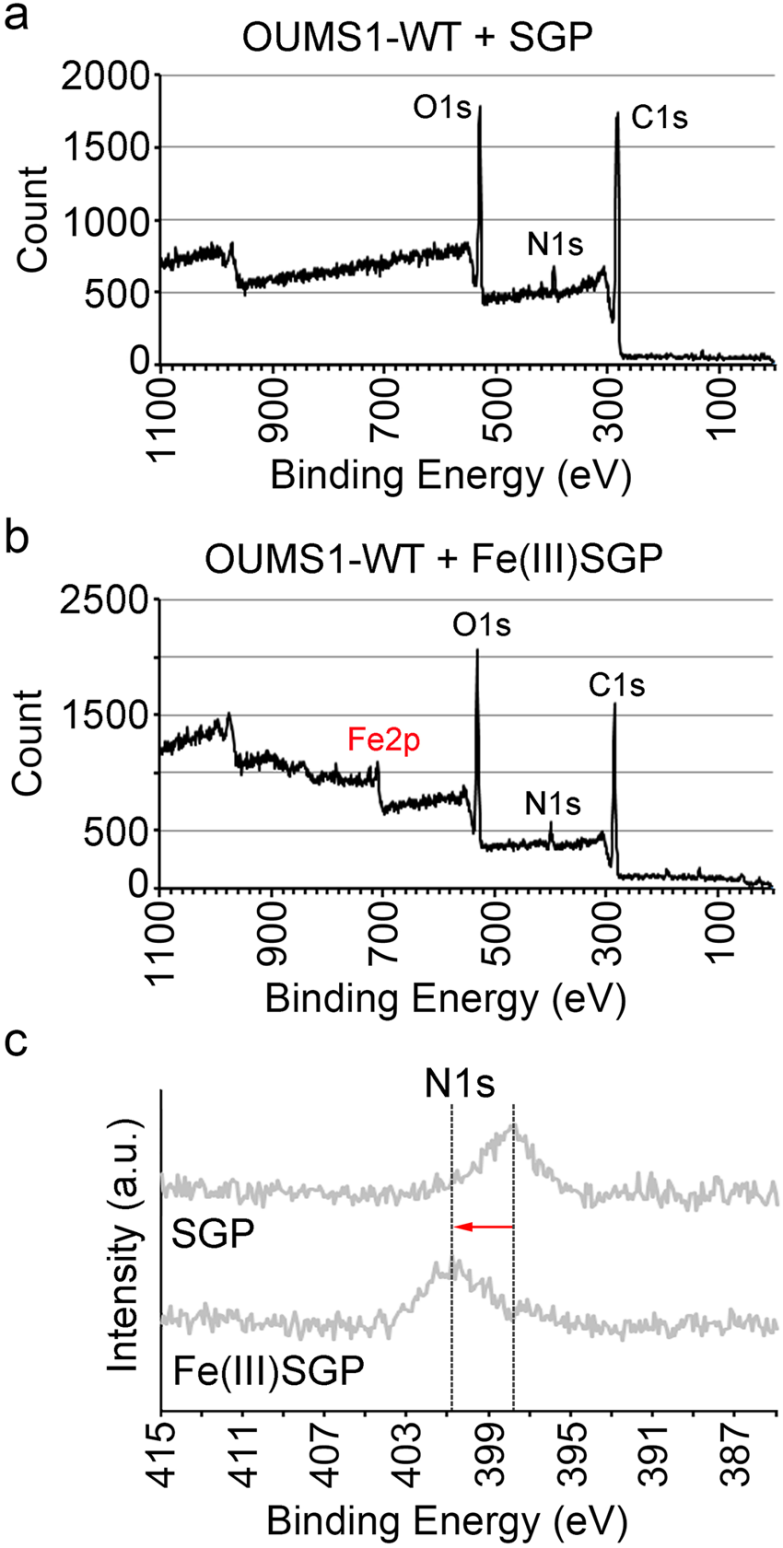
Photoelectron spectra acquired by XPS from OUMS1-WT sheaths incubated in SGP or in Fe(III)SGP. SGP: (**a**) Peaks of C1s, O1s, and N1s detected. Fe(III)SGP: (**b**) Additional Fe2p peaks detected. (**c**) Shift of N1s peak toward higher binding energy.

Since XPS can determine the intrinsic binding energy of the atomic orbital, which shifts chemically with the surroundings of the atom, the electronic state of the material surface was analyzed using XPS^29^. From the XPS measurement of SGP-incubated OUMS1-WT sheaths, peaks of O1s (532 eV), N1s (399 eV), and C1s (284 eV) were detected, suggesting the organic nature of the sheaths (Fig. 5a). Additional peaks of Fe2p (710 and 723 eV) were detected in the sheaths incubated in Fe(III)SGP (Fig. 5b). The N1s peak yielded by Fe(III)SGP-incubated sheaths shifted toward a binding energy higher than that yielded by the SGP-incubated sheaths (Fig. 5c), suggesting that Fe(III) minerals affected the N-related functional group such as NH_2_^30, 31^.

### Binding energy shift on the surface of NH_2_-holding C-polymer and NH_2_-coated polystyrene beads caused by incubation with Fe(III)SGP

**Figure 6:**
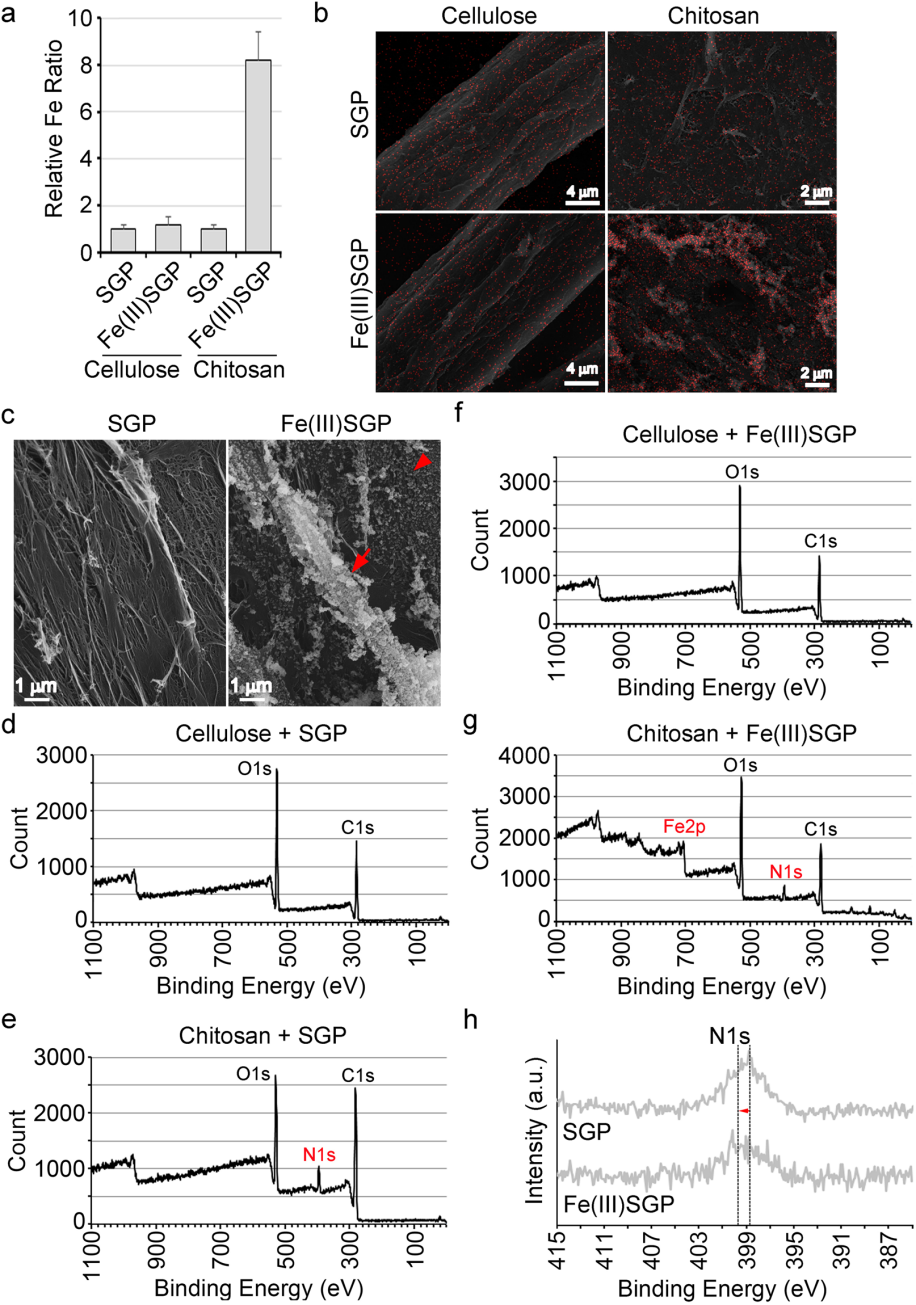
Behavior of Fe(III) minerals on cellulose and chitosan. (**a**) Fe ratios on cellulose and chitosan after incubating in Fe(III)SGP relative to At% of Fe = 1 in Fe-free SGP. (**b**) Top, merged images of SEM and EDX Fe distribution on cellulose and chitosan incubated in SGP. Bottom, Merged images of cellulose and chitosan incubated in Fe(III)SGP, showing Fe deposition on chitosan. (**c**) Left, SGP-incubated chitosan without any fine particles attached. Right, Fe(III)SGP-incubated chitosan with abundant fine particles (arrowheads), especially on aggregated fibrils (arrows). (**d**,**f**) XPS spectra of cellulose mixed with either SGP or Fe(III)SGP; note O1s and C1s peaks but none for N and Fe. (**e**,**g**) XPS spectra for chitosan after incubation in either SGP or Fe(III)SGP; note O1s, C1s, and N1s peaks in SGP incubation, additional Fe2p peaks in Fe(III)SGP. (**h**) N1s peak shifted toward higher binding energy after Fe(III)SGP incubation.

The described results led us to consider that NH_2_ in the OUMS1-WT sheath skeleton plausibly contributes to encrustation of the skeleton with Fe(III) minerals. We verified this possibility in model experiments to compare the affinity of cellulose (β-glucose polymer) and chitosan (GlcN polymer) and that of NH_2_-coated polystyrene beads and uncoated (plain) beads for Fe(III) minerals. The Fe ratio of chitosan increased drastically after incubation in Fe(III)SGP relative to At% of Fe = 1 in Fe-free SGP, while that of cellulose after the incubation in SGP was comparable to that in Fe(III)SGP (Fig. 6a). SEM and EDX indicated that aggregated Fe(III) minerals were attached to the chitosan fibrils after incubation, while the smooth surfaces of cellulose fibers were unchanged after incubation with SGP and Fe(III)SGP (Fig. 6b,c). In XPS, peaks of photoelectron O1s and C1s were detected for cellulose, while an additional N1s peak was detected for chitosan (Fig. 6d–g). The incubation in Fe(III)SGP yielded additional peaks of Fe2p for chitosan, but not for cellulose (Fig. 6f,g), suggesting the possible binding of Fe(III) minerals to NH_2_ of chitosan. Similar to the results for OUMS1-WT sheaths, the binding energy of N1s in chitosan shifted to a higher level (Fig. 6h), suggesting that NH_2_ of chitosan was influenced by Fe(III) minerals.

**Figure 7:**
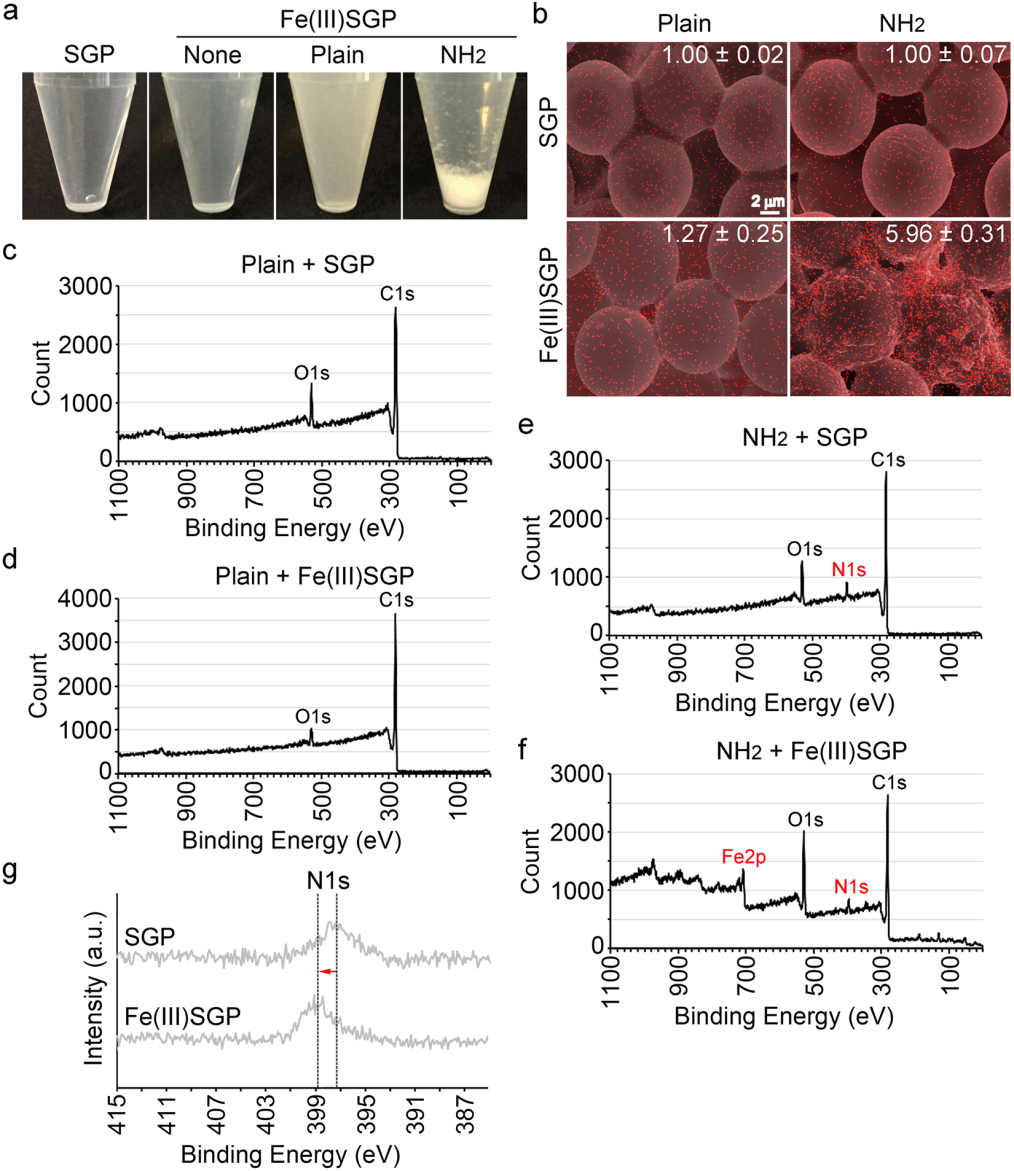
Behavior of Fe(III) minerals on plain and NH_2_-coated polystyrene beads. (**a**) Precipitation only on NH_2_-coated beads (NH_2_ beads) within 30 min after incubating in the suspension of Fe(III) minerals. (**b**) Top, merged images of SEM and EDX Fe distribution on plain and NH_2_ beads incubated with SGP. Bottom, merged image of the respective beads incubated with Fe(III)SGP, showing Fe deposition on NH_2_ beads. Fe ratios in Fe(III)SGP detected by XRF relative to At% of Fe = 1 on SGP-incubated beads: ~1.3 for plain beads, ~6.0 for NH_2_ beads. (**c**,**d**) XPS spectra of plain beads incubated with SGP and Fe(III)SGP, respectively, detecting O1s and C1s but no N and Fe peaks. (**e**,**f**) XPS spectra of NH_2_ beads incubated in SGP and Fe(III)SGP, respectively, detecting O1s, C1s, and N1s with SGP incubation and additional Fe2p peaks with Fe(III)SGP. (**g**) N1s peak shifted toward higher binding energy after incubation in Fe(III)SGP.

To further confirm the involvement of NH_2_ with the Fe encrustation, we examined the affinity of NH_2_-coated polystyrene beads (hereafter NH_2_ beads) and uncoated beads (hereafter plain beads) for Fe(III) minerals. When NH_2_ beads were incubated with Fe(III)SGP, a precipitate formed within 30 min, while the suspension of plain beads remained turbid (Fig. 7a). SEM revealed that the surfaces of the NH_2_-beads after Fe(III)SGP incubation were heavily coated with granular or rod-shaped Fe particles in contrast to the smooth surface of the plain and NH_2_ beads after SGP incubation (Figs 7b and S3c). The Fe atomic percentage determined by XRF was ~6.0 for the NH_2_-coated beads and ~1.3 for the plain after incubation in Fe(III)SGP (Fig. 7b). XPS detected photoelectron C1s and O1s peaks derived from polystyrene in plain beads and an additional N1s peak from the NH_2_ beads (Fig. 7c,e). The peak patterns from the plain beads in SGP and in Fe(III)SGP were comparable (Fig. 7c,d). In contrast, additional Fe2p peaks were detected in NH_2_ beads after incubation in Fe(III)SGP (Fig. 7e,f). Notably, incubation of NH_2_ beads in Fe(III)SGP shifted the N1s peak toward a higher binding energy (Fig. 7g), again suggesting an interaction between Fe(III) minerals and NH_2_. When acetylated NH_2_ beads (NH-Ac beads) were incubated with Fe(III)SGP, lack of Fe attachment to these beads was confirmed by SEM, EDX, and XPS (Fig. S3b–e).

## Discussion

The behavior of dissolved metals in natural bodies of water is strongly influenced by particular inorganic and organic materials^32^, suggesting complex interactions of various metal-complexing agents in aquatic systems with microbes and/or their constituent polymers. Generally, extracellular polymeric substances (EPS) of bacteria contain carbohydrates, proteins, lipids, extracellular DNA, and humic substances^33^, which possess various types of terminal functional groups in the molecules. The exopolymer fibrils of the *Leptothrix* sheaths are made of EPS^7, 15, 21, 22^, as such as they can also bind metals. The *Leptothrix* sheaths have a high affinity for a variety of metal cations such as Fe, Zn, and Pb^6, 21–25, 27, 28, 34–36^. Negatively charged functional groups of saccharic and proteinaceous materials in the *Leptothrix* sheaths and *Gallionella* stalks contribute to attracting dissolved cations^6, 7^. Several lines of evidence presented here indicate that NH_2_ in amino sugars and amino acids in the *Leptothrix* sheath is involved at least in direct deposition of Fe(III) minerals onto the sheath skeleton, which is composed of organic exofibrils. The Fe(III) minerals generated in this study are a complex of Fe oxyhydroxides^37^ and light elements such as P, S, K, Ca, and Cl from the surrounding medium (Fig. S1). Fe oxyhydroxides tend to be negatively charged below pH 7.0^38^. In addition, binding of Fe oxyhydroxides with the added P was proved to cause a more negative surface charge^39^. Therefore, we infer that the interaction between negatively and positively charged Fe(III) minerals and NH_2_, respectively, could be one of the driving forces for binding the minerals to *Leptothrix* sheath fibrils at pH 7.0 used in this study, although this assumption should be verified in the near future.

Note that we do not interpret our present results as meaning that NH_2_ is the only functional group that interacts with Fe(III) minerals, because masking of NH_2_ in sheaths with specific NH_2_-reactive reagents did not completely block the Fe(III) minerals deposition onto the immature sheaths (Fig. 3d), although the reagents appeared to block most of the terminal NH_2_ in the sheath skeleton. This incomplete blocking suggests the possible involvement of other functional groups in the deposition. On the basis of earlier reports^6, 21^, there is no reason to rule out possible synergistic reactions between NH_2_ and other functional groups such as COOH. At present, we infer that polymer-directed mineralization is a general phenomenon that could occur in any system containing NH_2_-enriched polysaccharides originating from an organism.

The energy shift that was detected in the OUMS1-WT sheaths, chitosan, and NH_2_ beads incubated with Fe(III) minerals (Figs 5–7) could be due to the influence of Fe(III) minerals as they approach the N-atomic orbital, which could readily shift chemically in the atom^29^. For example, the electron density around N is reduced when the covalent bond forms between NH_2_ and Fe(III) minerals or from the intense attractive force of charged particles^30, 31^. On the basis of earlier reports^30, 37^, we infer that in the aqueous phase the positive charge from NH_2_ and the negative charge from ferric oxyhydroxides may interact and that the chemical shift of N2p may reflect this interaction, although further detailed analyses are necessary to verify these and/or other possibilities such as a covalent bond, Van der Waals force, and condensation reaction of OH^−^.

Mineral precipitation could be an important determinant of microbial activity levels in the environment, and thus, spatially resolved analyses of organic compound combined with high-resolution mineralogical information should enhance our understanding of biomineralization mechanisms and promote the development of templated models to use in fabricating new materials^15^. Detailed information on the synthetic mechanism for biological organic–inorganic complexes will help expand the use of these ingenious complexes to benefit life. A variety of industrial applications such as lithium-ion battery anode material, catalyst enhancer, and pigment have been developed for metal-encrusted *Leptothrix* sheaths^7–10^. Current information on the roles of NH_2_ in Fe encrustation of the *Leptothrix* sheaths will greatly help in harnessing biomineralization mechanisms to create novel functional materials^26^. Toward creating novel materials, we should examine whether NH_2_-blocking affects the potential of other functional groups to bind inorganics including Fe, how NH_2_-blocking influences previously reported the functioning of sheath fibrils, and how we can artificially synthesize NH_2_-rich polymers with high reactivity to inorganics for use as adsorbents and/or catalysts.

Organic polymers play important roles in ecosystems by adsorbing biologically important elements^26, 40^. Fe cycling, mediated by microbiological oxido-reduction of the element, is a vital environmental process, both on the micro and global scales, and iron-oxidizing bacteria such as *Leptothrix* are significant facilitators of ferric iron reduction in ecosystems^6^. A step-by-step approach such as the present study should improve our understanding of the complex systems of Fe circulation.

## Methods

### Strains, medium, and culturing

*Leptothrix cholodnii* strain OUMS1 (NITE BP-860) (hereafter OUMS1-WT) and its sheathless mutant (OUMS1-SL) were used^27, 28^. Cells of OUMS1-WT and OUMS1-SL, obtained from frozen stock cultures, were independently streaked onto SGP agar plates^27^ and incubated at 20 °C for 7 days. Single colonies were transferred separately to 25 ml of SGP in 200 ml aluminum foil-capped Erlenmeyer flasks and cultured on a rotary shaker at 20 °C and 70 rpm. After 2 days, 1–5 ml of the cell suspension (adjusted to 10 cfu ml^−1^ by densitometry) was used to inoculate 25 ml of (i) SGP, and (ii) SGP containing 500 μM FeSO_4_ (SGP + FeSO_4_), or (iii) 100 ml SGP containing three small pieces of Fe plate (SGP + Fe plate). Samples were examined after 2 days of incubation. Viability of OUMS1-WT in these culture conditions was confirmed by live/dead staining^41^ after a 4-day culture (Fig. S1d). Specimens incubated in SGP + Fe plate were used for preliminary microscopic observations, and those in SGP or SGP + FeSO_4_ were mainly used for the following affinity tests and electron microscopy, unless otherwise stated.

### Abiotic preparation of Fe(III) minerals in SGP

Our previous work provided evidence that in a shaken solution of SGP + FeSO_4_, Fe(II) was oxidized to Fe(III) almost completely within 6 h^24^. In the present study, uninoculated SGP + FeSO_4_ was shaken similarly for 8–12 h to ensure generation of abiotic Fe(III) minerals. The suspension of Fe(III) minerals in SGP [hereafter Fe(III)SGP] was used for the affinity tests, as follows.

### Determination of amino sugar composition in protein-free OUMS1-WT sheath remnants

Protein-free sheath remnants were prepared according to a previous protocol^24, 25^. OUMS1-WT cells, pelletized from 4–6 l of a 2-day-old culture in SGP, were washed once with ultrapure water (H_2_O) and resuspended in 22.5 ml of the lysis solution containing 2.5 mM EDTA and 150–500 μg lysozyme (Sigma-Aldrich), and incubated at 37 °C for 0.5–2 h. Then 2.5 ml of 10 % w v^−1^ SDS was added, and the suspension was shaken for 0.5–2 h at room temperature. Thus-prepared sheath remnants were washed twice with H_2_O, then treated with proteinase K (50–100 μg ml^−1^; Nacalai Tesque, Kyoto, Japan) at 37 °C for 12 h. The final protein-free sheath remnants were washed with H_2_O six times and analyzed for sugar composition as follows.

The sheath remnants (20 mg) were vacuum-dehydrated at 50 °C for 2 h, followed by gas phase hydrazinolysis at 110 °C for 1 h with Hydraclub S-204 (J-OI Mills, Tokyo, Japan) to release oligosaccharides from the remnants. The resultant product was dissolved in 1 ml of 0.2 N sodium bicarbonate (NaHCO_3_, pH 6.7), followed by filtration (0.8 μm pore, Advantest, Tokyo, Japan) and subsequent overnight dialysis against 100 mM 0.2 N NaHCO_3_ using a dialysis membrane (Molecular Weight Cut-off 12,000–14,000, Wako Pure Chemical, Osaka, Japan). For N-acetylation and demineralization, the dialyzed, freeze-dried sample was combined with 250 μl of 0.2 M ammonium acetate (pH 6.7) and 25 μl of acetic anhydride at room temperature for 30 min. This step was repeated for another 30 min. With the gradual addition of 100 % ethanol, the sample was freeze-dried to eliminate both reagents, then dissolved in 200 μl of H_2_O, demineralized using an acilyzer G0 (Asahi-kasei, Tokyo, Japan), and freeze-drying. The dried specimens were treated in 1 ml of 2 M trifluoroacetic acid at 100 °C for 2 h for acid hydrolysis. Subsequently, the hydrolyzed specimens were dissolved in 200 μl of H_2_O. Ten microliters of the solution was freeze-dried, followed by complete dissolution in 100 μl of pyridine and then combined wtih 20 μl of *N*,*O*-bis(trimethylsilyl) trifluoroacetamide (BSTFA) and 2 μl of trimethylchlorosilane (TMCS) at 60 °C for 30 min for trimethylsilylation. The resultant specimens were subjected to GC/MS analysis (Clarus SQ8T, Perkin Elmer, Waltham, MA, USA). The mass spectra were compared with those of a standard sugar mixture (Glc, Gal, and GalA: Wako Pure Chem.; GlcA: Sigma-Aldrich; GlcN: Yaizu Suisankagaku, Shizuoka, Japan).

### Fluorescent labeling to mask NH_2_ in sheath skeletons

The fluorescent protein, allophycocyanin (APC)-conjugated reagent (Dojindo, Kumamoto, Japan) (APC-reagent) was used to confirm the existence of NH_2_ in sheath skeletons of OUMS1-WT (Fig. 3b). In parallel, OUMS1-SL, which does not excrete exofibrils within 2 days after incubation in Fe-free SGP, was used as a negative control for exofibril absence. OUMS1-WT and OUMS1-SL, which had been precultured in SGP for 2 days, were separately mixed with this reagent at dilutions from 10^−2^ to 10^−3^ and incubated at 37 °C for 1–12 h. After four washes in SGP, the specimens were observed microscopically. For monitoring their affinity for Fe(III) minerals, they were incubated with Fe(III)SGP for 2 days before observation. For XRF analysis, the Fe(III)SGP-incubated specimens were washed six times in H_2_O and freeze-dried.

Two other reagents that can mask NH_2_^42^ were similarly tested: sulfo-NHS-acetate (1–10 mM, Tokyo Kasei, Tokyo, Japan) and acetic anhydride (200 mM, Nacalai Tesque), were used for masking NH_2_ in the OUMS1-WT sheath skeleton. OUMS1-WT, precultured in SGP for 2 days, was incubated with either reagent at 37 °C for 1–12 h. After washing with SGP four times, the respective specimens were incubated in Fe(III)SGP for 2 days, then washed six times in H_2_O, freeze-dried, and analyzed by XRF. For TEM/EDX imaging, the incubated specimens were chemically fixed as described below.

### Reference experiments using polysaccharides and polystyrene beads

Chitosan (Wako Pure Chem.) was incubated in SGP or Fe(III) SGP at a final concentration of 4 mg ml^−1^ for 12–16 h to examine its affinity for Fe(III) minerals. For comparison, the affinity of cellulose (Nacalai Tesque) for the minerals was tested. After incubation in SGP or Fe(III)SGP, these polymers were washed at least six times in H_2_O, freeze-dried, and subjected to XRF analysis, SEM/EDX imaging, and XPS measurement.

Additionally, three types of polystyrene beads (plain, NH_2_-coated, and acetylated-NH_2_-coated: 10 μm diameter each) (Micro mod, Rostock, Germany) were incubated in SGP or Fe(III)SGP at a final concentration of 2.5 mg ml^−1^. After incubation for 12–16 h, the beads were washed twice in H_2_O to remove SGP or Fe(III)SGP, then washed four times in 100 % ethanol and allowed to air-dry. Either ethanol-suspended or air-dried beads were subjected to the following XRF analysis, SEM/EDX imaging, and XPS measurement. The NH_2_ on acetylated-NH_2_-coated beads is supposed to be blocked by the acetylation.

### Differential interference contrast (DIC) and fluorescence microscopic imaging

OUMS1-WT and OUMS1-SL cells that had been treated with APC-reagent or for live/dead staining were observed with a BX51 system microscope (Olympus, Tokyo, Japan) equipped with DIC optics and a fluorescence attachment with a mercury lamp: U-MWIG3 U-MWU2, and U-MBIW3 (530–550, 330–360, and 460–490 nm excitation and 580, 430, and 520 nm emission, respectively) and dichroic mirror units.

### Scanning and transmission electron microscopy

For SEM/EDX, the above-H_2_O-washed specimens were fixed with a mixture of 2.5 % v v^−1^ glutaraldehyde and 1 % w v^−1^ OsO_4_ in 0.1 M cacodylate buffer (pH 7.0), washed with H_2_O, dehydrated with an increasing series of ethanol solution (30 %, 50 %, 70 %, 95 %, and 100 %), *t*-butanol, and critical-point drying, then placed on carbon tape (Nisshin EM, Tokyo, Japan) on a stub. Freeze-dried sugar chains were similarly attached to carbon tape, as were ethanol-suspended polystyrene beads, then air-dried. Specimens were coated with platinum (ca 15 nm thick) using an ion-sputter (E-1030, Hitachi, Tokyo, Japan) and then observed with an SEM (S-4300, Hitachi) equipped with an energy dispersive X-ray spectrometer (EDX) (Genesis 2000, Amtek-Edax, Berwyn, PA, USA) at 15 kV.

For TEM, the above-treated specimens collected by centrifugation were fixed with a mixture of 2 % v v^−1^ glutaraldehyde and 2 % v v^−1^ paraformaldehyde in 0.1 M phosphate buffer (pH 7.4) at 4 °C overnight. After a 30 min buffer wash, the specimens were embedded in 3 % agar in the buffer. Small pieces of the agar block were post-fixed with 2 % w v^−1^ OsO_4_ in 0.1 M phosphate buffer (pH 7.4) for 1.5 h, then washed with the buffer. Then, the specimens were dehydrated in an increasing series of ethanol solutions (30 %, 50 %, 70 %, 95 %, and 100 %), treated with propylene oxide, then embedded in Spurr’s resin. Sections (70–80 nm thick) were cut using a ultramicrotome (Leica, Wetzlar, Germany) equipped with a diamond knife (Ultra 45° 3.0 mm, Diatome, Hartfield, PA, USA) and stained with uranyl acetate and lead solutions and observed with a TEM (JEM-2100F, JEOL, Tokyo, Japan) equipped with EDX at 200 kV. The semiqualitative distribution maps were calculated by the Cliff-Lorimer method^43, 44^ to confirm their location.

### X-ray fluorescence (XRF) analysis

To determine the atomic percentage of Fe in the test specimens, freeze-dried OUMS1-WT sheaths and sugar polymers and air-dried polystyrene beads were packed into small aluminum pans for elemental analysis with an Orbis micro x-ray fluorescence (XRF) analyzer (Ametek, Berwyn, PA, USA). Atomic percentage of any detected element with a standard error was expressed as the mean (±SE) of 10 spots.

### X-ray photoelectron spectroscopy (XPS)

For obtaining photoelectron spectra from OUMS1-WT sheaths, sugar chain polymers and polystyrene beads, XPS measurements were carried out using monochromatic A1-Kα radiation (*hv* = 1486.6 eV) as described previously^45, 46^. Briefly, specimens were spread on carbon tape (Nisshin EM) and vacuum-dried. In the spectroscopy apparatus, surface charges on specimens were neutralized by using a low energy flood gun (~5 eV). Before evaluating the shifts in binding energy of the N1s in the respective specimens (Figs 5c, 6h and 7g), the detected N1s values in Fe(III)SGP-treated specimens (Figs 5b, 6g and 7f) were compensated on the basis of the value of C1s of the respective SGP-treated specimens (284.3 eV) (Figs 5a, 6e and 7e), because the binding energy levels of all elements varied slightly between Fe(III)SGP- and SGP-treated specimens. The photoelectron O1s spectrum acquired from all specimens was negligible, because it was apparently derived from contaminated carbonate within the x-ray photoelectron spectroscopy vacuum chamber of the XPS device.

### X-ray diffraction (XRD) analysis

To examine the crystallinity of the Fe(III) minerals obtained from SGP + Fe plate or SGP + FeSO_4_, XRD patterns of ethanol-washed and dried Fe(III) minerals were analyzed using an RINT-2500HF X-ray generator (Rigaku, Tokyo, Japan) with Cu-Kα radiation (voltage: 40 kV; current: 200 mA) as described previously^47^. The freeze-dried specimens were fixed on a zero background sample holder and scanned continuously from 10° to 90° (2*θ* value) at a rate of 3° min^−1^. The XRD pattern of the zero background sample holder was also measured.

## Electronic supplementary material

Supplementary Information
