# Supplementary material for: Amino group in Leptothrix sheath skeleton is responsible for direct deposition of Fe(III) minerals onto the sheaths
Source: Sci Rep. 2017 Jul 26;7:6498. doi: 10.1038/s41598-017-06644-8 (PMC5529543; doi:10.1038/s41598-017-06644-8)
Supplement: Supplementary file 1 — Supplementary Information [file 41598_2017_6644_MOESM1_ESM.pdf]

Supplementary Information

Amino group in *Leptothrix* sheath skeleton is responsible for direct deposition of Fe(III) minerals onto the sheaths

Tatsuki Kunoh[1,2], Syuji Matsumoto[1,2], Noriyuki Nagaoka[3], Shoko Kanashima[4], Katsuhiko Hino[4], Tetsuya Uchida[2], Katsunori Tamura[1,2], Hitoshi Kunoh[1,2] & Jun Takada[1,2†]

Supplemental Figures

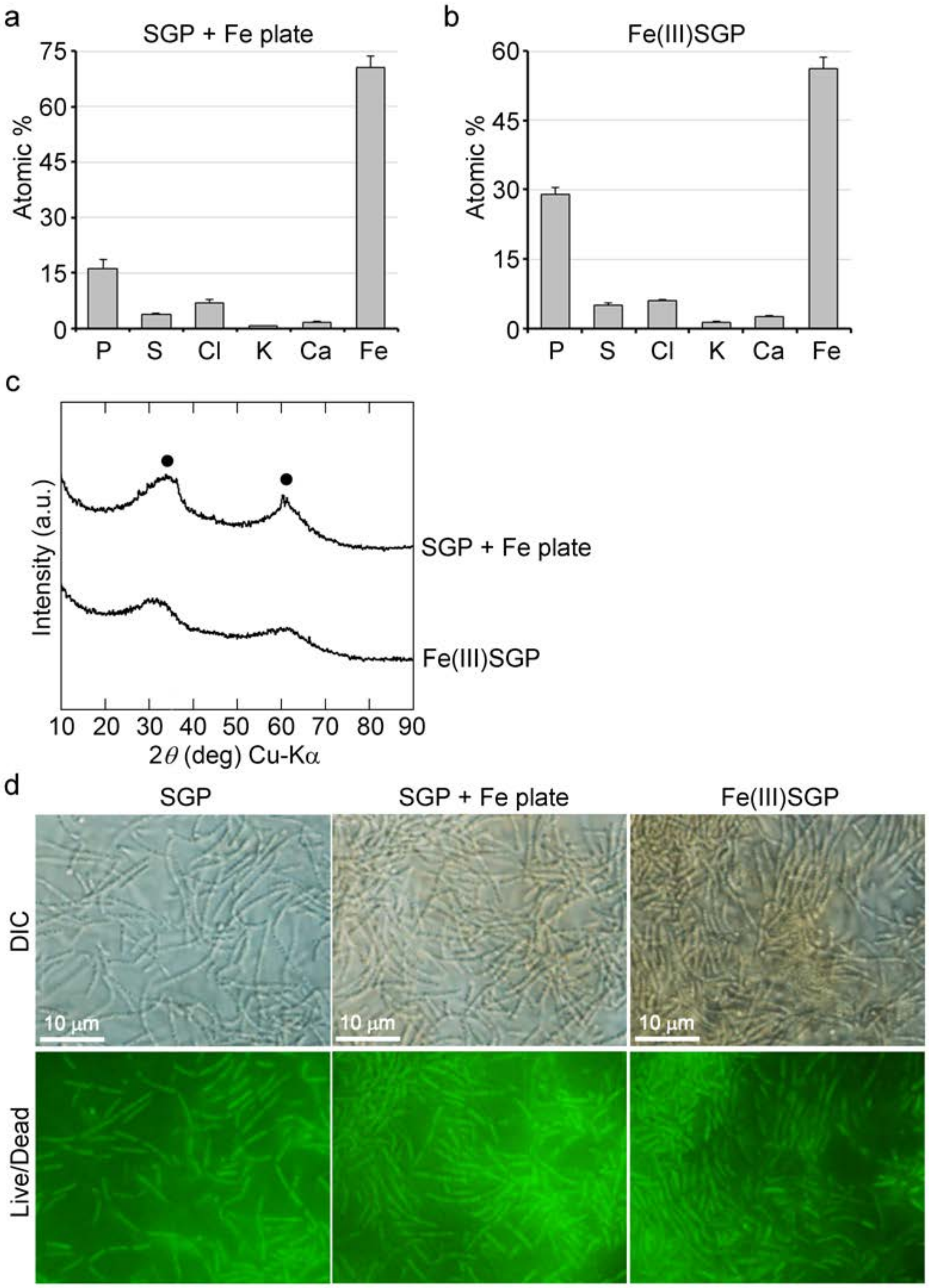

Figure S1. XRF (a, b) and XRD (c) analyses of abiotically generated Fe minerals after 2 days incubation in SGP+Fe plate and Fe(III)SGP and live/dead-stained images of cultured OUMS1-WT (d). a, b, Atomic percentages of elements in Fe minerals harvested from SGP+Fe plate and Fe(III)SGP, respectively, detected by XRF,

indicating the major element Fe is from the Fe source and minor elements from SGP components. **c,** XRD patterns showing 2-line ferrihydrite (circles) in both Fe minerals. **d**, DIC and fluorescent images of live/dead-stained OUMS1-WT cells cultured in SGP for 2 days (left) and in SGP+Fe plate and Fe(III)SGP an additional 2 days (center and right), respectively, showing the living state (green fluorescence) in these culture conditions.

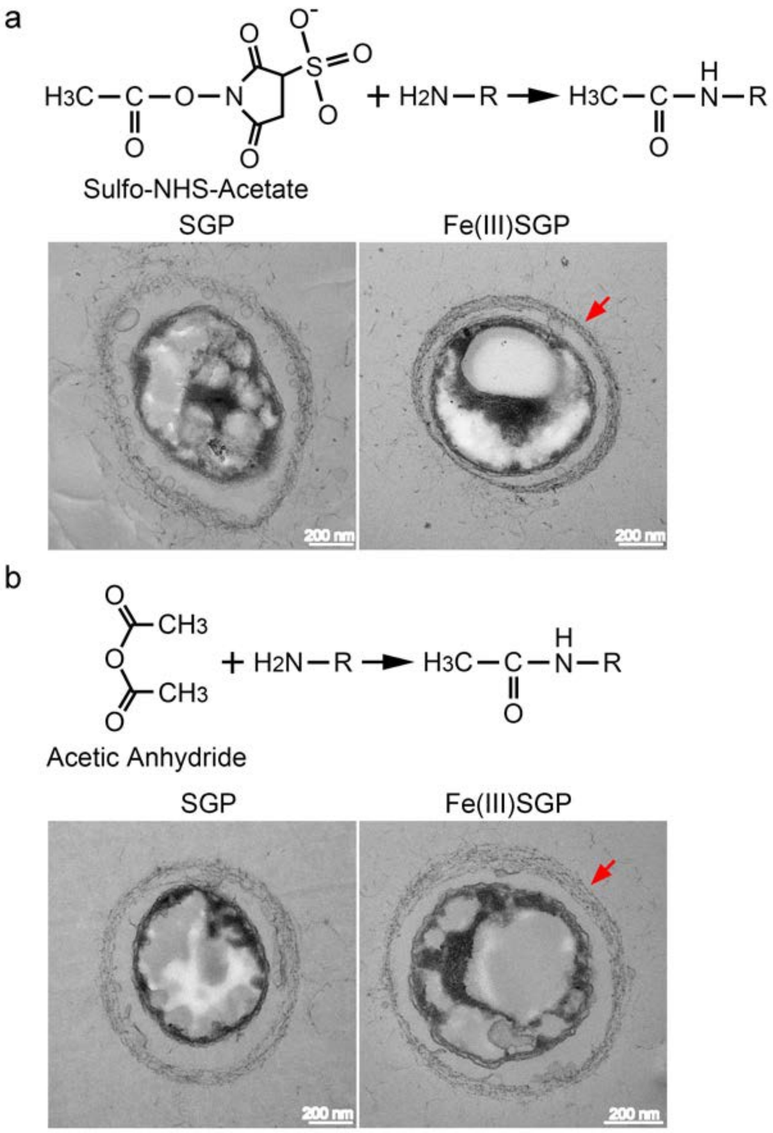

Figure S2. TEM image of sheath encasing OUMS1 cell with NH_2 masked with sulfo-NHS-acetate (a) or acetic anhydride (b). a, b, Top, schematic of respective binding of sulfo-NHS-acetate (a) and acetic anhydride (b) to NH_2. **a, b,** Bottom, thin, immature sheath (arrow) incubated in SGP or Fe(III)SGP. Note: no Fe was deposited in the NH_2-masked sheath even after incubating in Fe(III)SGP.

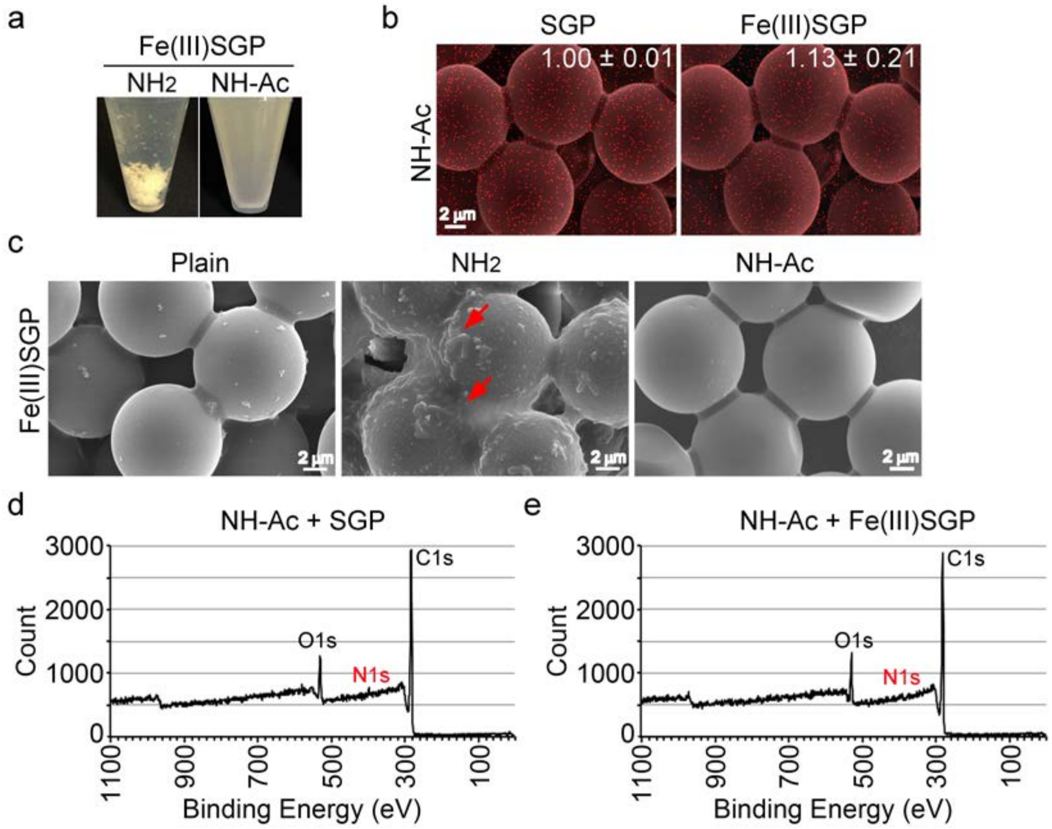

Figure S3. Behavior of Fe(III) minerals on plain, NH_2-coated, and acetylated NH_2-coated (NHAc) polystyrene beads. a, Precipitation of NH_2 beads, but not of NHAc beads, within 30 min after mixing with Fe(III)SGP. **b,** Merged images of SEM and EDX Fe distribution pattern on NHAc beads incubated in SGP or Fe(III)SGP. The XRF-detected relative Fe ratio on Fe(III)SGP-incubated beads was 1.13 relative to 1 on SGP-incubated beads. **c,** SEM images of plain, NH_2, and NHAc beads incubated in Fe(III)SGP. Note deposits of Fe minerals (arrows) only on NH_2 beads. **d, e,** XPS spectra of NHAc beads incubated in SGP or Fe(III)SGP. Note that Fe-related peaks were not detected from beads in either medium, and N1s peaks were too small for further analysis of any chemical shift.

Supplemental Table

**Table S1. Composition of silicon-glucose-peptone (SGP) medium**		
**Component**	**Amount (g l^{-1})**	**Concentration (mM)**
**Glucose**	**1**	**5.55**
**Soy peptone**	**1**	**ND**
**$Na_2SiO_3 \cdot 9H_2O$**	**0.2**	**0.7**
**$CaCl_2 \cdot 2H_2O$**	**0.044**	**0.3**
**$MgSO_4 \cdot 7H_2O$**	**0.041**	**0.17**
**$Na_2HPO_4 \cdot 12H_2O$**	**0.076**	**0.21**
**$KH_2PO_4 \cdot 2H_2O$**	**0.02**	**0.15**
**HEPES**	**2.38**	**10**
Medium was adjusted to pH 7.0 with 0.1 N NaOH, then brought to 1 liter with UPW.		
ND: not determined.		
